# Prevalence of and factors associated with contraceptive discontinuation in Kenya

**DOI:** 10.4102/phcfm.v14i1.2992

**Published:** 2022-05-24

**Authors:** Wambui Kungu, Alfred Agwanda, Anne Khasakhala

**Affiliations:** 1 Population Studies and Research Institute, Faculty of Social Sciences, University of Nairobi, Nairobi, Kenya

**Keywords:** contraceptive method, discontinuation, switching, abandonment in need, unintended births

## Abstract

**Background:**

The overwhelming uptake of contraception in Kenya at 58% suggests huge potential for a continued increase, but discontinuation threatens efforts to achieve new targets. Further increases in contraceptive prevalence will depend more on continuation and re-adoption amongst past users because unintended pregnancies would increasingly result from discontinuation. Eliminating discontinuations from side effects and method failure could increase continuation rates by 10%.

**Aim:**

To establish the prevalence and factors associated with contraceptive discontinuation.

**Setting:**

Kenya, with a successful family planning programme, but also the challenge of discontinuation rates of 31%.

**Methods:**

Contraceptive calendar data from the 2014 Kenya Demographic and Health Survey were used in the survival analysis approach.

**Results:**

Overall discontinuation rates were 37% (24 months) and 74% at (36 months), whilst discontinuation in need was 36%. Side effects accounted for 40% of discontinuations, whilst injection and pill recorded the highest rates. Current method emerged as a predictor of discontinuation at 24 months with the following hazard ratio (HR) at 95% confidence interval [CI]; intrauterine device (IUD) (HR = 0.466, CI = 0.254–0.857), injection (HR = 0.801, 95% CI = 0.690–0.930), implants (HR = 0.580, 95% CI = 0.429–0.784) and at 36 months, injection (HR = 0.808, 95% CI = 0.722–0.904) and implants (HR = 0.585, 95% CI = 0.468–0.730). Age (15–24 years) displayed influence only at 36 months (HR = 1.219, 95% CI = 1.044–1.424).

**Conclusion:**

The study showed a close link between contraceptive method used and discontinuation and thus the need to address method-related issues in an attempt to minimise discontinuation in Kenya. Expanding contraceptive options and improving the quality of service can scale up switching and thus help reduce discontinuation and unintended births.

## Introduction

Studies on discontinuation are important for family planning programme managers and policymakers as they may point to several problems within the programmes. Data on contraceptive discontinuation can help family planning programme managers improve the programmes to target women whose risk of unintended pregnancy is highest.^1^ Method-specific discontinuation rates from side effects may suggest problems in the information and counselling given when initiating or following up on a method. High switching between methods may imply dissatisfaction with the methods or availability and convenient access to a good method mix, whilst low switching may signify either satisfaction or limited access and/or method mix.^2^ Discontinuation attributed to failure may indicate incorrect use of a method, whilst high rates of discontinuation for reasons of access and availability may either imply challenges in supply and distribution of contraceptives or inadequate service delivery.^3^ Mostly, discontinuation is a result of reasons other than the desire for a child, and hence can be called premature.^4^

Previous studies on discontinuation have strongly associated contraceptive method choice with three different types of discontinuations, namely, abandonment in need, method failure and switching.^5^ The decision to continue or discontinue contraception involves factors such as how acceptable contraceptive options are, current and future situation and fertility desire. Evidence has shown that discontinuation was linked to low motivation to avoid pregnancy and that discontinuation because of quality-related issues was more common than discontinuation brought about by the reduced need for contraception.^6^ Consistent determinants of discontinuation include the method used, side effects, age, parity, fertility intentions, quality of care and changes in marital status.^7^ On the other hand, discontinuation is less consistently associated with the number of methods available, socio-economic factors, residence, partner’s disapproval, cost and accessibility of the method.^3,8^

Contraceptive discontinuation is associated with the quality of services as it is likely to affect contraceptive adoption and, more significantly, contraceptive continuation. Low discontinuation rates are seen as a measure of high-quality care, whilst high discontinuation rates imply poor-quality care. About 25% – 60% of discontinuations have been associated with quality-related reasons (all reasons apart from those of reduced need for contraception). Key determinants of discontinuation have been summarised as health systems elements, service quality and sociocultural factors.^9,10,11^

A close look at the contraceptive dynamics situation in countries in sub-Saharan Africa, such as Tanzania, Ghana and Ethiopia, reveals that over 30% of users discontinue contraception within the first 12 months and over 60% discontinue within 36 months for reasons such as side effects and inconvenience of the methods.^4^ This is a phenomenal concern for family planning programme managers as it waters down the various efforts to scale up the use of family planning methods.^9,12^ Side effects and health concerns are the leading reasons for discontinuation of modern methods. A study carried out in Pakistan found that elimination of the discontinuations caused by the side effects could increase contraceptive continuation rates (at 12 months) by 10%, whereas elimination of method failure could increase contraceptive continuation rates (at 12 months) by 6%.^13^

Discontinuation has far-reaching consequences on a country’s fertility, with up to 30% of unintended births resulting from contraceptive method failure.^14^ Data from developing countries have attributed 28% – 64% of total fertility rate (TFR) to discontinuations from reasons other than the desire for pregnancy.^15^ Studies have shown that as the level of the current use of contraception increases, continuity of use becomes an important measure of overall programme effectiveness because programme efforts generally shift from recruiting new users to satisfying current users and encouraging re-adoption amongst those who discontinued use.^9^ This implies that further increases in contraceptive prevalence rate (CPR) become more dependent on promotion of continuation rates and readoption amongst past users than on promotion of new acceptance rates because unwanted and mistimed pregnancies would rise from discontinuation of methods rather than from the failure to use contraception at all.^16^

Discontinuation has been given the analogy of a leaking bucket where some current contraceptive users slowly drop into another bucket of past and never users whose combination makes up unmet need, meaning the bucket of current users can never get full or even rise sustainably. It is therefore important to reduce the unmet need to discourage contraceptive discontinuation. In developing countries, up to 20% of unmet need results from discontinuation because of side effects.^17^

The recent overwhelming success in the uptake of family planning (FP) in Kenya of 58% CPR in 2014 shows huge potential for continued uptake in contraceptive prevalence but the challenge of retaining current users and attracting past users who want to reinitiate use remains. Evidence on which methods are discontinued more, why, after what periods and if clients switch methods when they are not satisfied with them can guide method counselling. The Kenya Demographic and Health Survey (KDHS) 2014 found high discontinuation at 31% within 12 months of initiating contraception, 18% unmet need for family planning and 35% unplanned births.^18^ Evidence has attributed 32% of recent unintended births in Kenya to discontinuation.^16^ It is therefore important to identify the factors that influence discontinuation so that they can be addressed where possible and the high rates of discontinuation can be minimised.

The main objective of this study was to establish the prevalence and factors associated with contraceptive discontinuation.

## Research methods and design

### Study design

This study extracted secondary data from the 2014 KDHS and specifically the contraceptive calendar data contained in the woman’s questionnaire.

### Setting

The study setting was Kenya, a country that has had a very successful family planning programme since the 1980s but is faced with the challenge of a contraceptive discontinuation rate of 30%. The KDHS covers the whole country. The extracted data included all the women who reported current use of a modern method of contraception at the time of interview during the KDHS 2014.

### Inclusion and exclusion criteria

The cases included in the extracted data were all those that had a date of beginning and a date of termination at 24 and 36 months before the KDHS interview date, respectively. The study analysed episodes of use from the major modern contraceptive methods: injection, pill, implants and intrauterine device (IUD). Those with zero episodes of use and those using sterilisation were excluded from the analysis. Cases of any ever use (as opposed to current use) of modern methods, which started before the last five years, were included to have a larger sample as long as there was a start date to the episode of use. Only cases of discontinuations where the reason for discontinuation was not blank in the five years preceding the survey were retained.

### Data source

The KDHS 2014 is the data source for this survey. It was a national household survey that provided data for various monitoring and impact evaluation indicators in population, health and nutrition. It involved collection of primary data using three different questionnaires: household, man’s and woman’s questionnaires. This study used the woman’s questionnaire, which targeted women aged 15–49 years and collected the 5-year retrospective contraceptive history of the woman through a contraceptive calendar. The contraceptive calendar in the demographic and health survey (DHS) survey woman’s questionnaire contains two columns: births, pregnancies and contraceptive use; and reasons for discontinuation of contraceptive. The information is presented in a string format. The calendar in KDHS 2014 required contraceptive information from the women for the last 60 months from the date of interview, that is, from May 2009 to around July 2014, depending on the month of interview as the survey spanned from May 2014 to July 2014. The KDHS woman’s data set is a rectangular data set having individual women aged 15–49 years as the unit of analysis.

### Data extraction

The following flow chart shows the process of data extraction from KDHS woman’s file to episodes of modern contraceptive use file.

The unit of analysis in the study was episodes of use and not the individual woman as shown in the diagram. Therefore, one woman may be represented by several episodes of contraceptive use in the KDHS contraceptive calendar. When extracting data from the calendar, a contraceptive events-based data set was created wherein each episode of contraceptive use represented one observation or a case. This was performed through the creation of separate variables for each month of the calendar. The list of variables created and their codes are outlined as follows: EV900 – Date episode begins in century months (CMC), EV901 – Date episode ends in CMC, EV901A – Duration of episode, EV902A – Specific episode code, EV903A – Specific discontinuation type code, EV906A – Marital status at the end of episode, EV902 – Episode code (numeric), EV903 –Discontinuation code (numeric), EV904 – Prior episode code, EV904X – Duration of prior episode, EV905 – Next episode code, EV905X – Duration of next episode, TYPEDISC – Type of discontinuation and METHODISC – Method discontinued.

The contraceptive methods and reasons for discontinuation that were in the form of strings were converted into numeric codes using the specific position of the code in the string. This was done by assigning each reason a label and a code. Then months of continuous use for the same woman were put together and aggregated to become single episodes of use for different methods. Switching a method translated to a new episode.

The main challenge in converting the woman’s data file to a format that can be used for the analysis was converting the string months of use into the units of the data set so that every month of use could become a single case. After that a file with episodes of continuous use of modern contraceptives that had a start date and an end date that fell within the last 60 months was created.

### Data analysis

This study analysed the duration of time from initiation of a method of contraception to when one stopped using it (discontinuation), hence the event of interest was discontinuation. The variables of the start and end date of each episode of use already exist in KDHS data set. A survival variable, duration of the event was created to be the dependent variable at 24 and 36 months duration of contraceptive use.

Discontinuations were examined by reason, type of discontinuation, method of contraception and socio-demographic variables (age, education, wealth status, place of residence and type of region) to establish the levels and determinants of contraceptive discontinuation. The unit of analysis in the study was an episode of modern contraceptive use, which is defined as a period of continuous use of a particular method of contraception in months. Types of discontinuation were categorised into three: switching, discontinuation when in need and other (where method failure was combined with discontinuation when not in need). Lifetables produced the descriptive results, discontinuation rates, whilst the regression model used was the Cox proportional hazards. The key parameter in Cox regression is the hazard ratio, which is a measure of effect that compares two or more groups in predicting the outcome variable. The Statistical Package for Social Sciences (SPSS) version 22 was used to conduct the analysis. Data used were weighted for more accuracy. The DHS has computed and recommended a weighting variable for data from the Women’s questionnaire, which weighs variable 005/1 000 000.

## Results

The number of episodes observed at 24 months was 2368 from 2102 women, whilst at 36 months, 2851 women contributed to 3355 episodes.

### Distribution of discontinuation episodes

The first stage of analysis identified the distribution of the discontinuation episodes according to the three categories of discontinuation types, namely, switching to another method, discontinuation whilst still in need and other. Figure 2 presents the results of the distribution.

**FIGURE 1:**
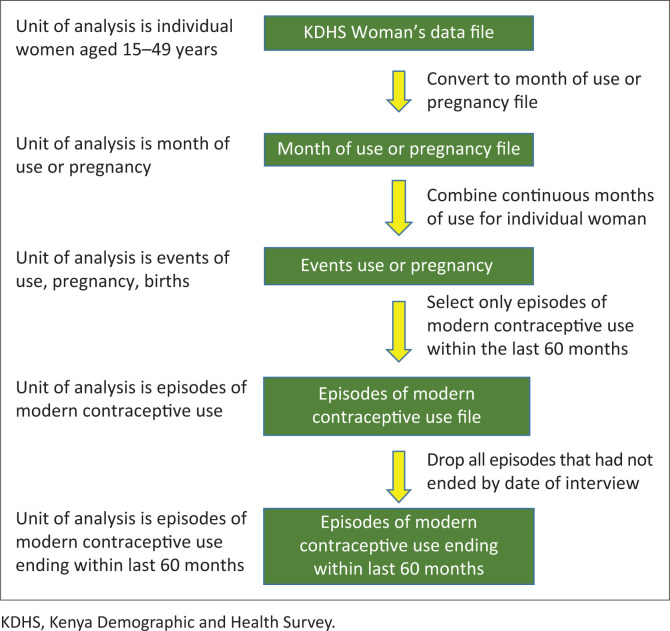
Process of creating a modern contraceptive use episodes’ file from the KDHS woman’s file.

**FIGURE 2:**
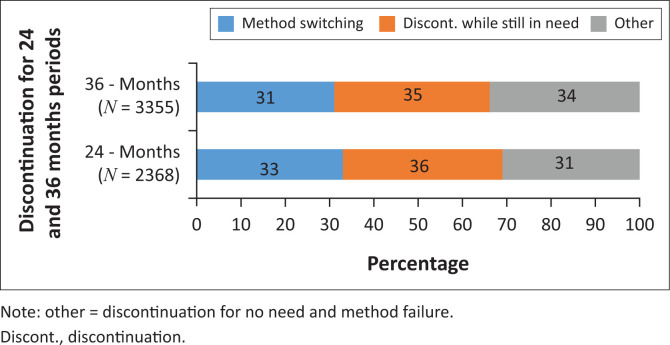
Percentage distribution of discontinuation episodes by type of discontinuation for 24 and 36 months periods, respectively.

Data in Figure 2 indicate that there is no real significant difference in discontinuation by type for both time durations.

### Overall discontinuation rates

The issue of method discontinuation rates was of particular interest in the study because of the close association between discontinuation and method type. The overall discontinuation rates were calculated at 12-month intervals from 12 to 36 months, respectively, and the results are shown in Table 1.

**TABLE 1:** Overall discontinuation rates for specific methods at 12, 24 and 36 months, respectively.

Method	12-month discontinuation rates (%)	24-month discontinuation rates (%)	36-month discontinuation rates (%)	Number of episodes of use (*n*)
Pill	18	18	46	973
IUD	8	15	36	86
Injection	15	30	42	2058
Implant	12	25	42	238
**All methods**	**30**	**37**	**74**	**3355**

IUD, intrauterine device.

The obtained rates of discontinuation at 12, 24 and 36 months are 30%, 37% and 74%, respectively. Pill users have the highest overall propensity to discontinue at all periods followed by users of injection. Users of the IUD were least likely to discontinue use.

Table 2 presents the percent distribution of discontinuation of use by contraceptive method and characteristics of the respondents for 24 and 36 months.

**TABLE 2:** Distribution of contraceptive use discontinuation episodes by selected variables for 24 and 36 months of contraceptive use, respectively.

Variable	24 months (weighted)		36 months (weighted)	
	Episodes	Percentage	Episodes	Percentage
**Method**				
Pill	690	29.1	973	29.0
IUD	60	2.5	86	2.6
Injection	1429	60.3	2058	61.3
Implants	189	8.0	238	7.1
**Age (year)**				
15–24	493	20.8	667	19.9
25–34	1291	54.5	1808	53.9
35–49	584	24.7	880	26.2
**Education**				
None	1388	60.2	1963	60.5
Primary	686	29.8	953	29.4
Secondary +	230	10.0	330	10.2
**Residence**				
Rural	1375	58.1	1944	57.9
Urban	993	41.9	1411	42.1
**Wealth status**				
Lower	706	29.8	1029	30.7
Middle	528	22.3	758	22.6
Higher	1134	47.9	1568	46.7
**Region**				
Low contraceptive	652	27.5	948	28.3
High contraceptive	1716	72.5	2407	71.7

**Total**	**2368**	**100.0**	**3355**	**100.0**

IUD, intrauterine device.

As expected, discontinuation rates varied by the contraceptive method. The distribution of discontinuation by the type of method shows users of injection were more likely to discontinue within 24 or 36 months compared with users of other methods. The two methods took up a combined share of 90% of the episodes showing that discontinuation is very prevalent amongst their users. Implants and IUD had the lowest discontinuation rates

When the discontinuation was examined by different characteristics, a higher propensity to discontinue the use of any method occurred amongst women aged 25–34 years. Users with no education were also more likely to discontinue the use of any of the methods for both periods of analysis. Women residing in regions with high contraceptive prevalence showed higher likelihood to discontinue the use compared with users in regions with lower contraceptive prevalence.

### Reasons for discontinuation

**FIGURE 3:**
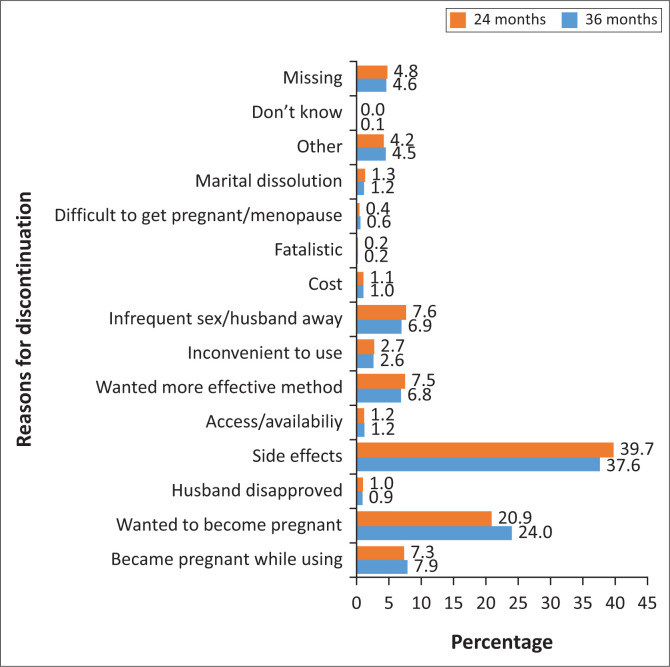
Percentage distribution of discontinuations in the 24 and 36 months preceding the survey by the main reason for discontinuation, Kenya 2014.

This section presents reasons for discontinuation (Figure 3). The main reason was side effects at 39.7% for 24 months and 37.6% for 36 months followed by wanted to become pregnant at 20.9% (24 months) and 24% (36 months). Method failure and ‘wanted more effective method’ accounted for about 7.0% each for both durations, respectively.

### Differentials in discontinuation by types

A cross-tabulation of discontinuation types by the different method types and variables was then conducted as presented in Table 3.

**TABLE 3:** Percentage distribution of discontinuation types by selected variables for 24 and 36 months of contraceptive use.

Variable	24 months† (weighted) (%)			36 months‡ (weighted) (%)		
	Switching	Discontinuation whilst in need	Other	Switching	Discontinuation whilst in need	Other
**Method**						
Pill	40.6	28.6	30.8	38.6	28.7	32.7
IUD	41.7	26.7	31.6	41.9	26.7	31.4
Injection	29.5	38.8	31.7	26.7	38.6	34.7
Implants	30.7	39.2	30.1	30.3	37.8	31.9
**Age (year)**						
15–24	25.4	38.6	36.0	23.4	39.2	37.4
25–34	32.9	34.5	32.6	29.9	33.9	36.2
35–49	40.1	35.1	24.8	38.3	35.3	26.4
**Education**						
None	33.7	37.4	28.9	30.7	38.2	31.1
Primary	34.8	32.9	32.3	33.3	31.8	34.9
Secondary +	29.1	27.4	43.5	29.7	24.8	45.5
**Residence**						
Rural	35.1	36.3	28.6	31.9	36.4	31.7
Urban	30.3	34.5	35.2	29.2	33.9	36.9
**Wealth status**						
Lower	24.5	44.2	31.3	21.0	45.1	33.9
Middle	37.1	35.2	27.7	33.9	35.1	31.0
Higher	36.6	30.3	33.1	35.7	29.1	35.2
**Region**						
Low contraceptive	24.1	45.9	30.0	21.8	44.8	33.4
High contraceptive	36.6	31.6	31.8	34.3	31.6	34.1

IUD, intrauterine device.

† , *N* = 2368.

‡ , *N* = 3355.

### Switching

Switching is defined as a change from one method to another by a user. About 42% of IUD users were more likely to switch to another method followed by users of the pill at 40%. Similarly, users of injection and implants had rates of about 30% each. The chances of switching to another method increased with age. Users who were over 35 years were more likely to switch to other methods (39% of the cases), whilst younger women were less likely to switch to other methods. There were no major differences by level of education in the chances of switching from one method to another.

Women living in rural areas were more likely to switch to other methods irrespective of the duration of use. Users from the regions where contraceptive prevalence was high were more likely to switch to other methods compared with users in regions where contraceptive prevalence was low. Those users who came from households categorised with middle and higher wealth index were more likely to switch to other methods.

### Discontinuation (abandonment) whilst in need

This group represents users who for one reason or another are likely to discontinue the use of contraception but still want to prevent pregnancy (in need). Users of injection and implants had the largest shares at 39% each, followed by the pill at 29% average. Women in the age group of 15–24 years had the largest share of discontinuing the use whilst still in need of contraception compared with other age groups. The share of women in the ‘no education’ category was also more likely to discontinue the use of contraception whilst still in need compared with women in other education categories.

There were no significant differentials in abandonment amongst urban and rural residences for both time durations. Large differences were seen in the proportion of women who discontinue the use of contraception whilst still in need according to regions categorised as high or low contraceptive prevalence and by household wealth status.

### Determinants of discontinuation

This section presents the chances of discontinuation of use controlling for other factors. This was done using Cox proportional hazards regression model that considers the duration of use. The hazard rate represents the potential per unit time of an individual discontinuing a method in a given episode of use. The results are presented in Table 4.

**TABLE 4:** Factors influencing discontinuation after 24 and 36 months duration of contraceptive use.

Variable	24 months† (weighted)			36 months‡ (weighted)		
	*p*	HR	95% CI	*p*	HR	95% CI
**Method**						
Pill	Ref	1.000	Ref	Ref	1.000	Ref
IUD	0.014	0.466*	0.254–0.857	0.099	0.680	0.429–1.076
Injection	0.004	0.801*	0.690–0.930	0.000	0.808*	0.722–0.904
Implants	0.000	0.580*	0.429–0.784	0.000	0.585*	0.468–0.730
**Age (Year)**						
15–24	0.247	1.131	0.918–1.392	0.012	1.219*	1.044–1.424
25–34	0.904	1.012	0.835–1.227	0.824	1.016	0.883–1.169
35+	Ref	1.000	Ref	Ref	1.000	Ref
**Education**						
None	Ref	1.000	Ref	Ref	1.000	Ref
Primary	0.560	1.049	0.894–1.230	0.953	0.997	0.886–1.121
Secondary+	0.265	1.147	0.901–1.458	0.756	0.972	0.811–1.164
**Residence**						
Urban	Ref	1.000	Ref	Ref	1.000	Ref
Rural	0.971	1.003	0.858–1.173	0.531	1.038	0.924–1.165
**Region**						
High contraception	Ref	1.000	Ref	Ref	1.000	Ref
Low contraception	0.189	0.900	0.768–1.054	0.732	1.020	0.909–1.146
**Wealth**						
Lower	Ref	1.000	Ref	Ref	1.000	Ref
Middle	0.275	0.897	0.737–1.091	0.657	1.033	0.896–1.190
Higher	0.464	0.931	0.769–1.127	0.753	0.978	0.852–1.123

IUD, intrauterine device; Ref, reference category; CI, confidence interval; HR, hazard ratio.

* , *p* < 0.05.

† , *N* = 829.

‡ , *N* = 1490.

The regression analysis identified two variables that significantly determine discontinuation: method of contraception (IUD, injection and implants) and age (15–24 years). Age had no statistical significance on the likelihood of discontinuation at 24 months but significantly influenced the hazard of discontinuation at 36 months, whilst the use of the IUD had a significant effect on the hazard of discontinuation at 24 months but not at 36 months.

The study results showed that users of the IUD had their hazard of discontinuation reduced by half at 24 months when compared with pill users (hazard ratio [HR] = 0.466, 95% confidence interval [CI] = 0.254–0.857). The use of injection emerged as a very strong predictor of discontinuation at both durations, with injection users having 20% lower hazard of discontinuation than pill users: 24 months (HR = 0.801, 95% CI = 0.690–0.930) and 36 months (HR = 0.808, 95% CI = 0.722–0.904). Implants exhibited a very strong statistical significance on discontinuation at both durations. Implant users had their hazard of discontinuation decreased by 42% when compared with pill users at both durations: 24 months (HR = 0.580, 95% CI = 0.429–0.784) and 36 months (HR = 0.585, 95% CI = 0.468–0.730). The results showed that the use of the injection, IUDs and implants was significantly associated with a reduced hazard of discontinuation compared with the use of the pill.

Younger ages were associated with an increased hazard of discontinuation. Statistical significance emerged at 36 months in the 15–24 age category. Those aged 15–24 years had 1.2 times increased hazard of discontinuation compared with those aged 35+ years (HR = 1.219, CI = 1.044–1.424). The group thus had a 20% increase in the hazard of discontinuing a method when compared with the older 35+ category. Therefore, from the results it is clear that a change of contraceptive method or increasing age of a user would significantly influence (increase or reduce) the hazard of discontinuation.

There are two key functions in survival (event history) analysis such as discontinuation that were exhibited in this study. The first one is the hazard or distribution function, which is the potential per unit time to discontinue a method in the next episode and it shows the event of focus, that is, the hazard of discontinuation. The second is the survival function that shows the probabilities of survival, that is, not experiencing discontinuation or the proportion of users who continue to use the method. The survival function indicates the probability that the event of interest (discontinuation of use) has not yet occurred by a certain time *t* (duration of use). The probability of continuing to use at any point in time is a logarithmic decay (the rate of decrease decreases until it gets to zero). As for this study, the cases selected and included in the model were those that all had a date of beginning and a date of termination of contraceptive use – at the beginning the probability was 1. The results showed that the probabilities of survival (continuation) at given months were 6 (50%), 12 (35%), 24 (10%) and 36 (0%). Thus, the risk of discontinuation (failure) was exponentially increasing with time, from the start time to the end time of 36 months. The analysis showed a logarithmic decay to the maximum period of 36 months where the probability of survival of use for any specific modern contraceptive method was zero.

## Discussion

The overall rates of discontinuation of 30%, 37% and 74% for 12, 24 and 36 months, respectively, and the method-specific rates observed were consistent with the findings of other studies.^19^ Discontinuation rates for all methods were high at 36 months, which may signal spacers discontinuing because of a desire for pregnancy. Overall and method-specific discontinuation rates comparable to the results of this study were reported for KDHS 2003 data.^20^

Average switching rates of 32% were also consistent with other studies and suggest that discontinuation is not necessarily negative. Some women discontinue because of side effects, difficulty in use or inconvenience to them and their partners but switch to more suitable or effective methods.^9,21^ Lower rates of switching have been reported in other parts of Africa.^22^

However, it is not always the case that those who switch move to more effective methods. A study of developing countries found that 50% of women who discontinued switched to other modern methods, whilst 12% initiated traditional methods. About 25% did not start any method whilst 12% became pregnant. Women who used hormonal methods (which are more prone to side effects) for long periods were more likely to switch to non-hormonal methods.^23^

The study results showed switching was associated with increased age and lower rates were seen amongst the 15–24 years old women whilst higher rates emerged amongst the over 35 years old cohort. This was expected as younger women use contraception less and cautiously because most of them are not in long-term relationships and have no children.^24,25^ Other studies have associated older age with less likelihood of switching.^12^

Women with primary education showed more switching rates probably because they are the majority contraceptive users in Kenya (60%).^26^ Women with no education were more likely to use long-term methods, hence the lower switching rates. Those with a secondary education, because they are more exposed to contraceptive information and services, choose a more suitable method from initiation of contraception and thus were more likely to be satisfied with the methods they were using. Education has been found to be strongly associated with switching in several countries.^5^

Rural–urban differentials did not show any significant influence on discontinuation despite their significance in the choice of a method. Wealth categories did not show any significant association with discontinuation rates but large differentials based on residence and wealth were reported in previous studies.^5,27^ There were wide variations in the switching rates for lower contraceptive regions (22%) and higher contraceptive regions (34%), which is consistent with other studies.^5^ Women in higher contraceptive use regions are likely to have more exposure to contraceptive information and services and a wider method mix.

The greatest concern in discontinuation is those who stop contraception whilst still in need as they risk unintended pregnancies, and hence unwanted and mistimed births. This study showed 36% in this category and the rates were largely consistent in the bivariate results. However, women of lower wealth and women from lower contraceptive regions had much higher rates of discontinuation, at 45% and 50%, respectively. This may be explained by their tendency to use short-term methods that are prone to higher discontinuation rates and also their lower exposure to contraceptive information and services.^28^ The women who discontinue despite not wanting to get pregnant are exposed to unintended pregnancies, which might have negative outcomes, such as unsafe abortion and maternal and child morbidity and mortality, and may reverse the good progress so far realised in maternal and child health indicators.^29^

The leading cause of discontinuation was side effects, at almost 40%. According to information from KDHS, only 60% of current modern contraceptive users had been informed about the potential side effects of their chosen method and only 52% had been told how to handle them.^18^ Lower rates of 22% for side effects were reported by several countries.^4^ The high rates of discontinuation belie issues of service quality and differ from the high satisfaction levels of 97% reported by family planning exit clients in Kenyan health facilities.^30^

Discontinuations appeared to increase with the duration of time, with those who discontinued because of a desire to get pregnant being 14% at 24 months and 24% after 36 months. This is normal for users who are spacing births and KDHS 2014 found 26% of women wanted to get pregnant after 60 months of use.^18^

Discontinuations by method revealed that injection was the leading method followed by the pill. The KDHS 2014 results for 12 months of use placed the pill first in discontinuations and injection second.^18^ Injection being the most popular method in Kenya would naturally have more discontinuation episodes and at 24 and 36 months, most users would probably be discontinuing to get pregnant. It is more often used by women in age group of 25–34 years who are mostly spacing, hence the higher discontinuations. There could also be unintentional discontinuation in instances where women get late for re-injection or find the method unavailable during revisits.^31^ Injections are also hormonal methods that tend to have more issues with side effects and consequently discontinuations. Other studies in sub-Saharan Africa have found discontinuation rates for the injection to be normally low because the method is mostly used for long periods, but in other regions, discontinuations are higher.^32^ Injection, despite being a short-term method, is more convenient to use as adherence does not depend on the user and hence it has much lower discontinuation rates than the pill. Also, the higher discontinuations for both pills and injection could be explained by side effects and health concerns that are common to the two methods.^33,34^

Pills method is mostly used by women in the 35+ age category who are limiting births and exiting the reproductive age, hence the higher discontinuations.^33^ This is probably because the risk of becoming pregnant from missing to take the pill is of less concern to them than to younger women. Injections and implants that are not user-dependent are currently the leading methods of contraception in Kenya in that order.^26^

Implants and IUDs are long-acting reversible contraception (LARC), which are preferred by those who are limiting births or are not intending to get pregnant for several years, and hence their discontinuation rates are lower.^35,36^ This study’s findings are consistent with other studies that method continuation is better with longer-acting methods, such as implants and IUDs, that do not rely on user behaviour.^3,37^ Discontinuing the IUD calls for a proactive step to go for removal unlike the injection or pills, and as such, women who are more prone to discontinue may also be less likely to initiate the IUD.

Variations in rates of discontinuation by the method may point to user characteristics and the features of the method, whilst variations in rates of discontinuation by user characteristics may indicate the choice of methods for the users. There may be a connection between acceptance of a contraceptive method and its discontinuation rate with the use of the method. This relationship determines contraceptive continuity.^28^ This study thus focused on this link between the method choice and discontinuation.

With regard to age, the 15–24 age category had the lowest discontinuation rate as expected because their use of contraception is lower as they are just initiating sexual relationships or entering marriage. This has been reported in several studies.^12^ The 25–34-year-old women are the majority users of contraception as they are spacers and also they could be experimenting and switching methods as they seek the most suitable methods for their needs. This may explain more discontinuations amongst them. In other studies, however, younger women have been found to exhibit much higher discontinuation rates than older women and are more likely to discontinue whilst still in need of contraception than older women.^38^ Very high rates of discontinuation (60% in 6 months) amongst under 25 years age groups have been recorded in some countries.^34^

The results on education showed fewer episodes of discontinuation amongst women with primary and secondary education, which might be explained by their better understanding of contraception from higher exposure to contraceptive information and services, and hence they may be more settled on the method chosen. Similar results had been reported previously.^39^

More discontinuation episodes were seen in rural areas and low-contraceptive regions. A possible explanation is less access and exposure to contraceptive information and services.^7,40^ Women of higher wealth also exhibited much higher episodes of discontinuation than their middle and lower counterparts at 46% possibly because of better access to information and services, hence experimentation.

In the regression analysis, three contraceptive methods and age were found to influence discontinuation. Users of IUD had less hazards of discontinuation than users of pill at 24 months, whilst injection and implants had reduced hazard of discontinuation in comparison with the pill at both 24 and 36 months. Injection and implants are, respectively, the first and second most popular methods in Kenya, and hence the result is not unusual, whilst IUD is mostly used by those who want to limit births.

The contraceptive method selected is a significant predictor of discontinuation as reported in the discontinuation rates for various methods, especially hormonal ones. Women who used a method to limit their families significantly used it longer than those who used the method for child spacing.^10^ Method characteristics have been shown to influence discontinuation rates directly, by their influence on the risk of discontinuing a method, and also indirectly because they influence the choice of a particular method.^3^ Also, users of pills and injections may be more likely to discontinue than users of other methods.^12^

Age was also found to be a predictor of discontinuation in this study, especially for the 15–24 years age category. Similarly, age has emerged as a predictor of discontinuation in several studies, with younger women below 20 years of age exhibiting higher risks of discontinuation than older users. Younger women, 15–24 years age, are mostly in short-term relationships and experiment more with different methods, and hence prefer using short-term methods that are prone to higher discontinuation rates.^41^ However, a Bangladeshi study found that women aged between 25 and 34 years are more likely to discontinue than women who are less than 20 years old. Age is a very strong determinant of contraceptive method choice in both short-term and long-term methods because contraceptive intentions of spacing or limiting, which influence the choice of the method, vary significantly with age, hence its influence on continuation and discontinuation.^42^

Age is a critical factor in discontinuation amongst adolescents and young women. A woman’s age at the time of discontinuation was found to be consistently associated with all three types of discontinuation. Older women were less likely to abandon whilst in need, experience failure or switch methods than younger women.^4,39^ Younger women had more hazards of discontinuation whilst still in need of contraception than older women.^43^ Age and parity are mostly correlated and either factor may be an indication of the other.^44^

Even though socio-economic factors are important in influencing the type of discontinuation, a weak association between them and discontinuation has been previously reported.^2,45^ This study complements this as it did not show either an increase or a decrease in the hazard of discontinuation in socio-economic factors of education, wealth and residence. Certain characteristics led women to choose certain methods, and hence there were many differentials based on the variables. Studies conducted elsewhere have found that socio-economic factors are significant in discontinuation.^27^

## What are the implications for Kenya?

Much implications that family planning policy and programme managers need to address arise from these results.

Method-specific discontinuation rates showed injection and pill registering the highest rates. They pointed to the common issue of side effects and that injection, popularity notwithstanding, poses serious health concerns to many of its users. In addition, side effects being the main reason for discontinuation at about 40% mean that four in every 10 women have health issues from contraception and it is a serious health and programme concern. Method-related reasons combined with side effects take over 50% share of discontinuations. The findings raise queries whether clients are using the appropriate methods for their reproductive needs, may not have been properly counselled or did not get their preferred method.

The overall switching rates of about 35% driven by rural areas, increased wealth and high contraceptive use regions are encouraging and suggest the availability of options for women to shift to more suitable and effective methods. On the negative side, lower switching rates for lower contraceptive regions point to outstanding issues of less access and exposure to contraceptive information and services. The IUD also recorded very high rates of switching at an average of 50%, which raises concerns because it is not a hormonal method. Issues of insertion and side effects appear to stalk the method and if addressed might alter the trajectory of IUD as a LARC. However, it is encouraging that its share of discontinuation in need is not as high.

Discontinuation whilst in need is very high at 36% and if not followed by switching exposes the women to unintended pregnancies, which pose serious challenges to maternal and child health indicators. For injection and implants, discontinuation in need is very high and side effects may be the reason. The implant is long-term from three years and questions arise as to why a user would discontinue the method whilst still in need.

Women of lower wealth categories and lower contraceptive use regions recorded lower switching but more discontinuation whilst in need. Expanded information, counselling, access and a wider method mix may thus help to reduce discontinuations in need and promote switching.

There were fewer discontinuations amongst women of higher education and wealth status. It emphasises that continued women empowerment may give better access to more contraceptive information and services so that women could make informed and suitable contraceptive choices from initiation and thus be less inclined to discontinuations.

In general, the results raise a concern about the retention of a big proportion of contraceptive users and question the quality of counselling services. They suggest that gains in contraceptive uptake so strenuously achieved may be rolled back if the challenge of discontinuation and the underlying factors are not addressed.

## Limitations of the study

A possible limitation of the study on the discontinuation analysis is the accuracy of the information provided by the respondents as the women were asked to estimate on dates of contraception events that occurred up to five years ago. This could result in estimation and rounding as most of the respondents tend to round off numbers normally to the nearest 5 or 10 (i.e. anything around 10 such as 8, 9, 11, 12 would be rounded off to 10 and the same for 20). Others would just indicate between 20 and 30 instead of giving an actual figure. This could lead to a slight deviation in accuracy of information collected because of time elapsed and normally the more time that has elapsed since the event, the more tendency to round off.

However, reported data on contraceptive use in Kenya and elsewhere have been proved to be quite reliable and calendar data collected in a retrospective way remarkably mirror data collected in periodic surveys and have not shown worrying over-reporting or under-reporting patterns. An assessment of the data used in this study did not show any signs of heaping or inconsistency.

## Conclusion

This study documented the high prevalence of discontinuation mostly driven by side effects. A very close link was found between contraceptive method choice and discontinuation, with discontinuation being widespread across methods but more prominent amongst the pill and injection users. The study showed that method continuation, in general, was better with longer-acting methods, hence expanding contraceptive options (especially LARC) and improving the quality of service on counselling or follow-up, and provider attitudes can help reduce discontinuation and hence unintended births. This study did not show the significance of socio-economic factors in influencing discontinuation.

Contraceptive discontinuation has adverse implications on the health and development of a woman, her family and country and needs to be minimised. Given Kenya’s Family Planning 2030 (FP2030) and International Conference on Population and Development at 25 years (ICPD25) renewed commitments on reproductive health and targets for reduced fertility, the findings may inform interventions to seal the continuously leaking bucket of contraceptive users by reducing discontinuation and unmet need. This can contribute towards increased modern contraceptive prevalence rate (mCPR) and reduced TFR.

## References

[1] Ali MM, Park MH, Ngo TD. Levels and determinants of switching following intrauterine device discontinuation in 14 developing countries. Contraception. 2014;90(1):47–53. 10.1016/j.contraception.2014.03.008 24792145

[2] Blanc AK, Curtis SL, Croft TN. Monitoring contraceptive continuation: Links to fertility outcomes and quality of care. Stud Fam Plann. 2002;33(2):127–140. 10.1111/j.1728-4465.2002.00127.x 12132634

[3] Ali M, Cleland J. Determinants of contraceptive discontinuation in six developing countries. J Biosoc Sci. 1999;31(3):343–360. 10.1017/S0021932099003430 10453247

[4] Ali MM, Cleland JG, Shah IH. Causes and consequences of contraceptive discontinuation: Evidence from 60 demographic and health surveys. Geneva: World Health Organization; 2012.

[5] Ali MM, Cleland J. Contraceptive switching after method-related discontinuation: Levels and differentials. Stud Fam Plann. 2010;41(2):129–133. 10.1111/j.1728-4465.2010.00234.x 21466113

[6] Curtis S, Evens E, Sambisa W. Contraceptive discontinuation and unintended pregnancy: An imperfect relationship. Int Perspect Sex Reprod Health. 2011;37(2):58. 10.1363/3705811 21757420 PMC3912075

[7] Rizvi F, Irfan G. Reasons for discontinuation of contraceptive methods among couples with different family size and educational status. J Ayub Med Coll Abbottabad. 2012;24(1):101–104. 23855108

[8] Blanc AK, Curtis SL, Croft T. Does contraceptive discontinuation matter?: Quality of care and fertility consequences. Vol. 3. Measure Evaluation Technical Report Series. No. 3. Chapel Hill: Carolina Population Center, University of North Carolina; 1999.

[9] Castle S, Askew I. Contraceptive discontinuation: Reasons, challenges, and solutions. New York, NY: Population Council. 2015.

[10] Birhane K, Keesbury J, Fantahu M. Early discontinuation of Implanon and its associated factors among women who ever used Implanon in Ofla district, Tigray, Northern Ethiopia. IJPSR. 2015;6(3).

[11] Hubacher D, Olawo A, Manduku C, Kiarie J, Chen P-L. Preventing unintended pregnancy among young women in Kenya: Prospective cohort study to offer contraceptive implants. Contraception. 2012;86(5):511–517. 10.1016/j.contraception.2012.04.013 22633247

[12] Mahumud R, Hossain G, Sarker A, Hossain N, Jahangir A, Khan A. Prevalence and associated factors of contraceptive discontinuation and switching among Bangladeshi married women of reproductive age. J Contracept. 2015;6:13–19. 10.2147/OAJC.S76070 PMC5683137 29386920

[13] Mahmood A, Naz SS. Contraceptive use dynamics in Pakistan 2008-09. Islamabad: Population Council; 2012.

[14] Bradley SE, Croft T, Rutstein SO. The impact of contraceptive failure on unintended births and induced abortions: Estimates and strategies for reduction. Calverton, MD: ICF Macro; 2011.

[15] Cavallaro F, Benova L, Owolabi O, Ali M. A systematic review of the effectiveness of counselling strategies for modern contraceptive methods: What works and what doesn’t. BMJ Sex Reprod Health. 2019;0:1–16. 10.1136/bmjsrh-2019-200377 PMC7569400 31826883

[16] Jain AK, Winfrey W. Contribution of contraceptive discontinuation to unintended births in 36 developing countries. Stud Fam Plann. 2017;48(3):269–278. 10.1111/sifp.12023 28398595

[17] Jain A. The leaking bucket phenomenon in family planning [homepage on the Internet]. 2014 [cited 2014 Sept 9]. Available from: http://champions4choice.Org/2014/09/The-Leaking-Bucket-Phenomenon-in-Family-Planning/#More-1429

[18] KNBS, ICF Macro. Kenya Demographic and Health Survey 2014 report [homepage on the Internet]. 2015 [cited 2022 Feb 11]. Available from: https://www.google.com/search?q=Kenya+Demographic+and+Health+Survey+2014+Report&rlz=1

[19] Ontiri S, Were V, Kabue M, Biesma-Blanco R, Stekelenburg J. Patterns and determinants of modern contraceptive discontinuation among women of reproductive age: Analysis of Kenya Demographic Health Surveys, 2003–2014. PLoS One. 2020;15(11):e0241605. 10.1371/journal.pone.0241605 33151972 PMC7643986

[20] Agwanda T. Contraceptive use dynamics in Kenya: Analysis of method choice and discontinuation. Nairobi: National Coordinating Agency for Population and Development.

[21] John R, Keesbury J, Hardee K. Trends in the contraceptive method mix in low- and middle-income countries: Analysis using a new ‘Average Deviation’ measure global health: Science and practice [homepage on the Internet]. [cited 2022 Feb 11]. Available from: https://www.ghspjournal.org/content/3/1/34.full 10.9745/GHSP-D-14-00199 PMC4356274 25745119

[22] Barden-O’Fallon J, Speizer IS, Calhoun LM, Corroon M. Women’s contraceptive discontinuation and switching behavior in urban Senegal, 2010–2015. BMC Womens Health. 2018;18(1):1–9. 10.1186/s12905-018-0529-9 29402320 PMC5800088

[23] Picavet C, Van der Leest L, Wijsen C. Contraceptive decision-making: Background and outcomes of contraceptive methods. Jakarta: Rutgers World Population Foundation (WPF); 2011.

[24] Ersek JL, Brunner Huber LR, Thompson ME, Warren-Findlow J. Satisfaction and discontinuation of contraception by contraceptive method among university women. Matern Child Health J. 2011;15(4):497–506. 10.1007/s10995-010-0610-y 20428934

[25] Kungu W, Agwanda A, Khasakhala A. Trends and determinants of contraceptive method choice among women aged 15–24 years in Kenya. F1000Research. 2020;9(197):197. 10.12688/f1000research.22481.1

[26] Kungu W. Contraceptive use dynamics in Kenya, 2003–2014 [thesis]. Nairobi: University of Nairobi; 2021.

[27] Agrahari K, Mohanty SK, Chauhan RK. Socio-economic differentials in contraceptive discontinuation in India. SAGE Open. 2016;6(2):2158244016646612. 10.1177/2158244016646612

[28] Magadi M, Curtis S. Trends and determinants of contraceptive method choice in Kenya. Stud Fam Plann. 2003 Sep;34(3):149–159. 10.1111/j.1728-4465.2003.00149.x 14558318

[29] Ministry of Health, APHRC, IPAS. The costs of treating unsafe abortion complications in public health facilities in Kenya. Nairobi: Ministry of Health; 2018.

[30] NCPD, World Bank, UNFPA. Kenya health service delivery indicator survey report, 2018 [homepage on the Internet]. 2019 [cited 2022 Feb 11]. Available from: https://sentaokenya.org/sdm_downloads/kenya-health-service-delivery-indicator-survey-report-2018/

[31] Baumgartner JN, Morroni C, Mlobeli RD, et al. Timeliness of contraceptive reinjections in South Africa and its relation to unintentional discontinuation. Int Fam Plan Perspect. 2007;33(2):66–74. 10.1363/3306607 17588850

[32] Mitchel MJ, Thistle P. Acceptability of levonorgestrel subdermal implants versus tubal ligation for long-term contraception in a rural population of Zimbabwe. Contraception. 2004;70(6):483–486. 10.1016/j.contraception.2004.05.004 15541410

[33] Cho MK. Use of combined oral contraceptives in perimenopausal women. Chonnam Med J. 2018;54(3):153–158. 10.4068/cmj.2018.54.3.153 30288370 PMC6165915

[34] Kamalifard M, Malkouti J, Pezeski M, Velayati A. Continuation and discontinuation reasons of LD contraceptives among Iranian women. Int J Womens Health Reprod Sci. 2014 Jul;2(5):287–290.

[35] Shoupe D. LARC methods: Entering a new age of contraception and reproductive health. Contracept Reprod Med. 2016 Feb 23;1(1):4. 10.1186/s40834-016-0011-8 29201394 PMC5675060

[36] Kungu W, Khasakhala A, Agwanda A. Trends and factors associated with long-acting reversible contraception in Kenya. F1000Research. 2020;9:382. 35673521 10.12688/f1000research.23857.1 PMC9152462

[37] Curtis SL, Blanc AK. Determinants of contraceptive failure, switching, and discontinuation: An analysis of DHS contraceptive histories. Calverton, MD: Macro International; 1997.

[38] Rocca CH, Harper CC, Raine-Bennett TR. Young women’s perceptions of the benefits of childbearing: Associations with contraceptive use and pregnancy. Perspect Sex Reprod Health. 2013;45(1):23–31. 10.1363/4502313 23489854 PMC3620026

[39] Irani L, Speizer I, Curtis S, Ongechi K. Impact of place of residence and household wealth on contraceptive use patterns among urban women in Kenya [home page on the Internet]. 2012.

[40] Mobolaji JW, Bisiriyu L, Bamiwuye SO. Contraceptive discontinuation among Nigerian women: Exploring the ethnic variations. Ife Res Publ Geogr. 2017;14(1):47–58.

[41] Bradley SE, Schwandt H, Khan S. Levels, trends, and reasons for contraceptive discontinuation. DHS Analytical Studies No. 20. Calverton, MA: Macro International Inc.; 2009.

[42] Kungu W, Khasakhala A, Agwanda A. Use of long-acting reversible contraception among adolescents and young women in Kenya. PLoS One. 2020;15(11):e0241506. 10.1371/journal.pone.0241506 33170851 PMC7654813

[43] Danielle S, Casner T, Secura GM, Peipert JF, Madden T. Characteristics associated with discontinuation of long-acting reversible contraception within the first 6 months of use. Obstet Gynecol. 2013;122(6):1214. 10.1097/01.AOG.0000435452.86108.59 24201685 PMC4051392

[44] Safari W, Urassa M, Mtenga B, et al. Contraceptive use and discontinuation among women in rural North-West Tanzania. Contracept Reprod Med. 2019;4(1):1–10. 10.1186/s40834-019-0100-6 31754451 PMC6852765

[45] Magadi M, Zulu E, Ezeh A, Curtis S. Contraceptive use dynamics in Kenya. Nairobi: African population and health Research, Calverton, MA: Macro International Inc. APHRC and MEASURE Evaluation Project; 2001.

